# Socioeconomic impacts of elimination of onchocerciasis in Abu-Hamed focus, northern Sudan: lessons after elimination

**DOI:** 10.1186/s13104-020-05101-6

**Published:** 2020-05-26

**Authors:** Ayman Ahmed, Anas Elbashir, Asgad A. Mohamed, Asha A. Alim, Asia Mubarak, Duaa Abdelrahman, Eilaf Mohammed, Nouh S. Mohamed, Arwa H. Elaagip, Isam M. A. Zarroug, Noma Mounkaila, Hanan Tahir

**Affiliations:** 1 Institute of Endemic Diseases, University of Khartoum, Khartoum, Sudan; 2 Public and Tropical Health Programmes, University of Medical Sciences and Technology, Khartoum, Sudan; 3 Department of Parasitology and Medical Entomology, Faculty of Medical Laboratory Sciences, Nile University, Khartoum, Sudan; 4 Department of Parasitology and Medical Entomology, Faculty of Medical Laboratory Sciences, University of Khartoum, Khartoum, Sudan; 5 Onchocerciasis Control/Elimination Programme, National Programme for Prevention of Blindness (NPPB), Federal Ministry of Health, Khartoum, Sudan

**Keywords:** Onchocerciasis, NTDs, Elimination, Socioeconomic, Abu-Hamed, Sudan

## Abstract

**Objectives:**

Onchocerciasis is one of the most devastating neglected tropical diseases and it is mostly prevalent in Africa. The disease has important heavy social and economic burdens on the infected populations including low productivity, unemployment, social isolation, and stigma. A cross-sectional study was implemented using a well-established questionnaire to investigate the socio-economic impacts of Onchocerciasis elimination in Abu-Hamed, River Nile State, Sudan in 2015; 512 participants in ten affected communities were interviewed.

**Results:**

Our findings revealed that these communities are recovering from the social and economic burden of the diseases. Ninety percent of the research participants reported general satisfaction about elimination of the disease in their community, 48.3% of them attended secondary school or university. Only 0.6% reported unemployment. Also, 25.3% and 24.7% of the participants were workers and farmers respectively. Except about the vector biting and nuisance, the majority of the respondents (90%) had no complain related to the disease after the elimination. Also, 90.5% of the participants reported either stable or increase in their work performance during the last 12 months. About 93.8% of the respondents were engaged in normal daily life activities and involved in happy events like marriage and giving birth during the last 12 months.

## Introduction

Onchocerciasis, commonly known as river blindness disease, is a parasitic disease that is caused by the worm *Onchocerca volvulus*. It is transmitted by the female black flies, mainly *Simulium damnosum sensus lato* [1]. Onchocerciasis is one of the major neglected tropical diseases (NTDs) and it is mainly prevailing in 31 African countries, 6 countries in Americas and one Asian country, Yemen, with more than 100 million people at high risk of infection globally [2]. The mature worm has long reproductive lifespan up to 11 years; during which it releases numbers of microfilariae (mf) daily in the human host body [3, 4]. The Onchocerciasis Control Programs are relying on Community Directed Treatment with Ivermectin (CDTI) distributing the freely donated ivermectin (Mectizan^®^) to the populations at risk annually or biannually [1, 5].

Onchocerciasis found in Sudan in four foci: Northern Sudan (Abu-Hamed focus, Northern State); Eastern Sudan (Galabat sub-focus, Gadaref state), South and Southwest Sudan (Radom focus, South Darfur state), and Southern Sudan (Khor Yabous, Blue Nile state) [6],. The Abu-Hamed focus is unique for being the northernmost Onchocerciasis focus in the world [6–9]. The control of Onchocerciasis in Abu-Hamed started with an annual community-based treatment with Ivermectin (CDTI) in 1998. In 2006, the Government of Sudan launched an Onchocerciasis elimination policy and switched from annual to semi-annual CDTI [10]. Comprehensive surveys showed low level of transmission in 2007 [11], and complete interruption of the disease transmission in 2011 that was officially declared by the Sudan government in 2012 [10, 12]. Following World Health Organization (WHO) guidelines and after a 3-year post-treatment surveillance, the elimination of Abu Hamed focus was officially declared in 2015 [13].

Information on the social and economic impacts of Onchocerciasis elimination are required to assess and evaluate the success and guide the redevelopment of the focus population [13]. The present study was designed to investigate the socio-economic impacts of Onchocerciasis on Abu-Hamed populations and assess the social changes in their lives after the elimination of the disease.

## Main text

### Methods

#### Study design and study area

**Fig. 1:**
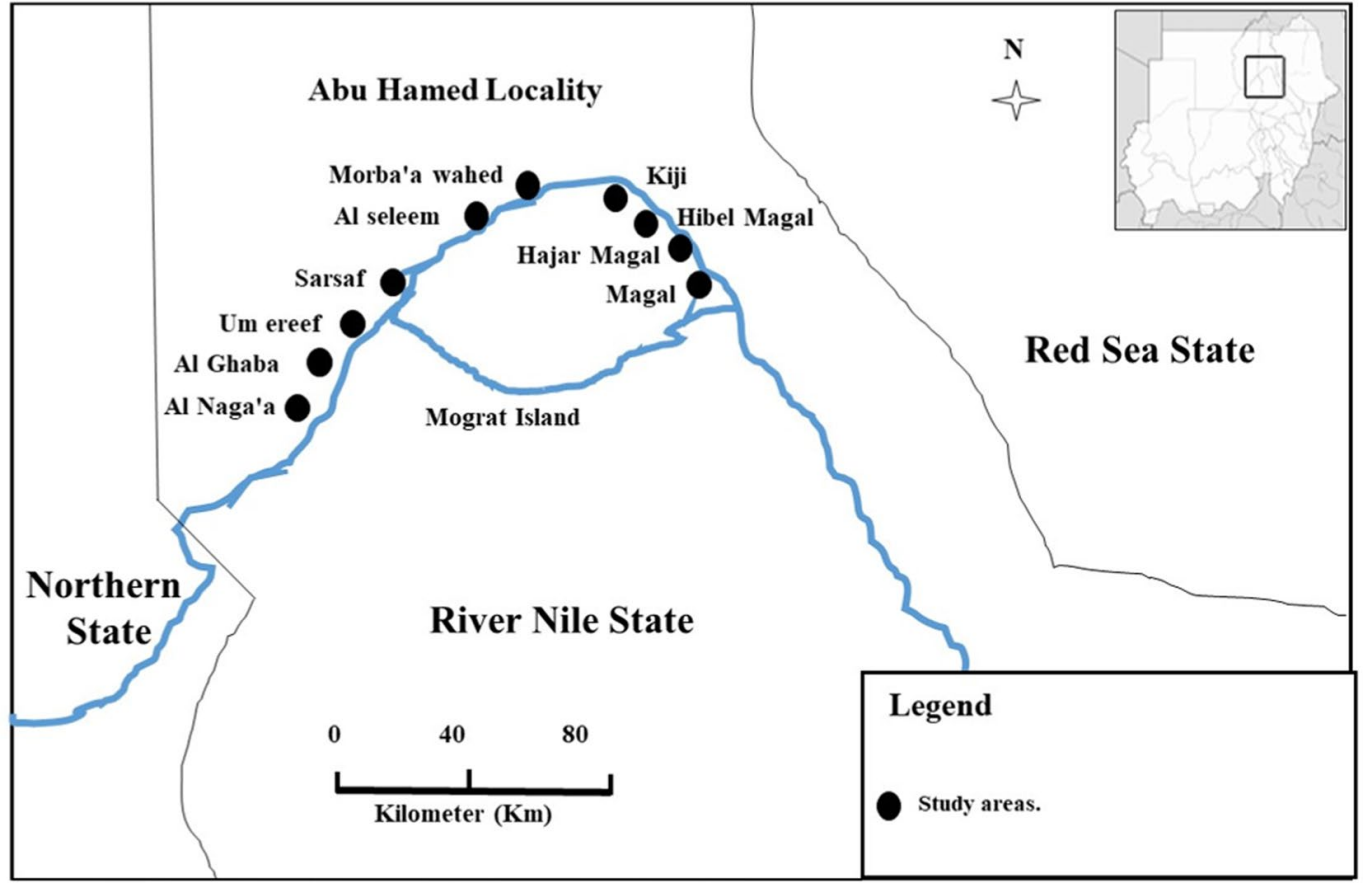
Map display the study sites location in the Sudan

This study was implemented in the period of September to October 2015, by the end of the 3-years post-treatment surveillance and the interruption of the disease transmission, soon after the official declaration of the elimination of the Onchocerciasis in Abu-Hamed. A cross-sectional study was implemented in Abu-Hamed area which is centered around the River Nile in the middle of the Nubian Desert (N 19° 32.49′–18° 17.29′, E 32° 13.86′–33° 55.00′) at an altitude range of 260–920 km. Sampling was done using a multi-stage technique; where 10 communities were randomly selected in Mograte district (Fig. 1), the last area that reported the disease in Abu-Hamed focus then the research participants were randomly selected in proportion to the size of the community, 10% household per community Adding 10% non-response rate the final sample size was 512 participants.

### Community survey

The research team was trained on interviewing research participants prior to the data collection. Data collection was done using a well-establish questionnaire (pre-developed and pretested by UMST), interviews were conducted by the trained research team. Each participant has been interviewed using his/her local language to entirely capture the demographic, socioeconomic, and environmental information.

### Statistical analysis

Demographical data, socioeconomic, and environmental information means, standard deviations and frequencies were analyzed using the Statistical Package for Social Sciences (SPSS) version 16.

## Results

### Sociodemographic characteristics of the study population

The study population (n = 512) were 12 to 89 years old with average age of 43 ± 16 years. The male-to-female was 1.7:1. The duration of stay in the respective communities varied from < 1 year to 89 years with an average period of living in the village of 35 ± 19 years. About 89% of the participants were living in their communities more than 10 years, and 82.5% of them did not travel out of their respective communities.

Regarding the education level of the participants, 12.4% did not attend any form of schooling. Adult schooling was attended by 2.4%, primary school by 36.1%, secondary by 38.7% and university by 9.6%. Also, unemployment was reported by 0.6% of the participants and 25.9% of the study participants were housewives. The main occupations in Abu-Hamed were laborer (25.3%), farmers (24.7%), and trading/commerce (12.1%). Teachers, drivers and students represented 2.9%, 2.5%, and 2.3% respectively. The remaining 4.3% included health professionals (1.9%), security personnel (0.8%), self-employed (0.8%), and fishermen (0.2%).

Regarding the main source of income, 28.0% did not have income. They were either housewives, students or unemployed. Daily or irregular income was recorded for labors (27.8%). Farming was the main source of revenue for 25.0%, commerce/trade or self-employment represent 12.4%. Regular monthly salary was the main source of income for 6.8%; they were either teachers, health professionals, or security forces. Most of the study population had heard about onchocerciasis (97%), and only 2.8% participants did not hear about the disease. Onchocerciasis is known in Abu-Hamed by several local names which were provided by 95% of the participants. Onchocerciasis or river blindness is locally named as Ama Aljoor, Night blindness, Kurtaib, Filaria, and Kuntaib. The vector transmitting Onchocerciasis (the *S. hamedense*) was identified as black fly by 77.4% of the participants. Surprisingly, the local name of the vector was the same of the disease but not reported in the same proportion. Sociodemographic characteristics of the study population were described in Table 1.

**Table 1:** Sociodemographic characteristics of the study population (n = 512)

Variable	Number	Percent	Variable	Number	Percent
Gender (n = 512)			Schooling (n = 510)		
Male	321	62.7	Yes	434	85.1
Female	191	37.3	No	65	12.7
Marital status (n = 504)			No answer	11	2.2
Married	424	84.1	Education level (n = 509)		
Single	73	14.5	Secondary school	197	38.7
Widow	4	0.8	Primary school	184	36.1
Divorced	3	0.6	None	63	12.4
Age (n = 509)			University	49	9.6
Mean (SD)	42.8	15.7	Adult schooling	12	2.4
Residence in the village (n = 505)			No answer	4	0.8
Mean (SD)	35.3	19.4	Main source of income (n = 500)		
Less than 10 years	58	11.5	No income	140	28.0
10 years and above	447	88.5	Daily/irregular income	139	27.8
Travel out of the community (n = 498)			Farming	125	25.0
Yes	85	17.1	Commerce/self-employed	62	12.4
No	411	82.5	Regular Salary	34	6.8
No answer	2	0.4	Knowledge of Onchocerciasis local name (n = 493)		
Onchocerciasis local name (n = 476)			Yes	470	95.3
Filaria	441	92.7	No	18	3.7
Kuntaib	28	6.0	No answer	5	1.0
Night blindness	3	0.6	Identifying the vector (n = 430)		
River blindness	2	0.4	Black fly	333	77.4
Ama Aljoor	1	0.2	Mosquito	52	12.1
Kurtaib	1	0.2	Fly	20	4.7
Knowledge of the drug distributed (n = 512)			Other	25	5.8
Yes	369	72.1	Onchocerciasis vector local name (n = 360)		
No	143	27.9	Kuntaib	281	78.0
Ivermectin distribution ongoing (n = 486)			Kurtaib	76	21.1
Yes	13	2.7	Filaria	2	0.6
No	473	97.3	Small insect	1	0.3

### Health issues and social impact related to onchocerciasis

Ninety percent of the study participants raised a common compliant in the additional notes section of the questionnaire about the vector biting and nuisance particularly during the winter season when they usually harvesting their farms and crops.

Only 61 participants identified the name of the drug used as Mectizan or Ivermectin^®^. However, 369 participants reported to know the drug and they described it well in terms of presentation and dose administrated by community-distributors. Also, 97.3% of the participants reported that ivermectin mass distribution was halted (by the end of project) in their respective communities. The remaining 2.7% who reported continuation of ivermectin distribution had not provided any reason justifying the continuation of the distribution.

Regarding the impact of Onchocerciasis elimination in Abu-Hamed, 90% of the study population did not have any complain after the elimination apart from the vector biting and nuisance; persisting itching was reported by 7.2%. Other complains were less than 3.5% and were mainly about the need for developmental project to increase the employability of the locals.

Changes in the performance of children attending school had not been noticed by 91.9% of the study population. For those who noted changes (6.2%), school attendance increased for 52.6%. The participants’ rate in school performance as excellent was 30%, and good was 30%. While fair and poor were 25.0% and 15.0%, respectively.

Work performance of the participants remained stable for 60.5%, and increased for 30%, and poor for 9.5%. On the other hand, the revenue of the participants remained stable for 60.9%; and increased for 19.4%. The remaining 19.7% reported a decrease of their revenue.

Socialization was measured as the frequency of receiving guests and/or getting involved in social events in the last twelve months. 44.8% of the participants received guest sometimes, 34.3% less often and 20.9% regularly.

When participants were requested to share their opinion regarding stopping ivermectin mass distribution, 78.2% thought the distribution should continue because of the health benefits of the drug such as deworming and preventing other skin infections. Only 21.8% was in favor of stopping ivermectin distribution. Out of 105 participants, 91.4% justified the halt of the distribution because of the program objectives were achieved, and failure of the program may justify the decision for the 8.6%.

In the overall, the social impact of Onchocerciasis elimination in the ten communities surveyed can be estimated with a general public satisfaction reported from most of the study participants (90%). Health issues and social impact related to onchocerciasis among the study participants were illustrated in Table 2.

**Table 2:** Health issues and social impact parameters

Variable	Number	Percent	Variable	Number	Percent
Complain since distribution of the drug was halted (n = 512)			Knowledge of people currently complaining about Onchocerciasis symptoms (n = 506)		
No complain	461	90.0	No	453	89.5
Itching	37	7.2	Yes	53	10.5
Irritation	13	2.5	Change in the work performance of participants (n = 377)		
Other	1	0.3	Stable	228	60.5
Receiving guests in the twelve past months (n = 373)			Increased	113	30.0
More often	167	44.8	Poor	36	9.5
Less often	128	34.3	Change in the revenue of participants (n = 371)		
Regularly	78	20.9	Stable	226	60.9
Opinion regarding halt of ivermectin distribution (n = 482)			Decrease	73	19.7
Distribution to continue because of health benefits	377	78.2	Increase	72	19.4
Programme reached objectives and as halted	96	19.9	Receiving guests in the twelve past months (n = 373)		
Distribution to continue because of programme failed	9	1.9	More often	167	44.8
Change in school attendance (n = 19)			Less often	128	34.3
Increase	10	52.6	Regularly	78	20.9
Decrease	7	36.8	Social events in the last twelve months (n = 162)		
Stable	2	10.6	Birth	93	57.4
Change in school performance (n = 20)			Marriage	59	36.4
Good or better	12	60.0	Divorce	9	5.6
Fair or worse	8	40.0	Separate	1	0.6

## Discussion

The Sudanese Onchocerciasis Control/Elimination Program initiated the CDTI in Abu-Hamed with annual treatment in 1998 then followed by 4 years of dual treatment per year from 2007 to 2011. By 2012, the disease was interrupted and the treatment was stopped [10]. We conducted this study at the end of 2015 to assess the socioeconomic impacts of this success, and evaluated the community recovery from this disease. And the fact that Abu-Hamed is the most remote focus of Onchocerciasis in the north worldwide with a very limited opportunity of re-establishment of the infection after the elimination [13], this situation provided us with an extraordinary opportunity to study the socioeconomic impacts of the elimination of Onchocerciasis in Abu-Hamed focus.

Abu-Hamed focus of Onchocerciasis was identified for the first time by Morgan in 1958 [9], while the vector was reported in the area 5 years earlier [14]. Clinical studies revealed that this focus was entirely dominated by the dermal form of the disease and no case of the eye form has been reported from this area ever. It might be due to the unique strain of *O. volvulus* circulating in this area [7, 15], or the unique sub-species of the vector, *S. hamedense,* which is totally confined to the area [16].

Our results showed high rates of recovery at different aspects of the affected population. This is underscored by the relatively high number of new comers in the area, immigrate within the last 10 years are a good indicator for the re-nourishment of the area, since people usually forced to desert their homes and villages, and leave to avoid the infection [17, 18]. Although the percentages of our respondents enrolled had achieved different low level of education. However, this was in accordance with the average educational level of rural areas in Sudan [19]. Remarkably, few participants reported unemployment which is much lower than the country overall unemployment rate [20]. Also, the working participants, reported improvement or stability in their work performance leading to sustainable income and reducing the risk of poverty. There was fluctuation in the students’ school attendance. We perceived that as an improvement in their health situation associated with elimination because it correlates with areas most affected by the disease. Suggesting that these students are contributing in the family work to generate income [17, 21].

Majority of the participants had no complain about the disease, and the rest complained about persisting itching, social or mental stress either about themselves or someone they knew. This mainly due to previous infection, and they have been referred to proper health and mental care to intervene and help them overcoming their issues. This low percentage might be due to the population engagement with the control program activities which increased their awareness about the disease and reduced the level of stigma [22, 23].

Although there was no specific question about the vector biting or nuisance in the original questionnaire because the Onchocerciasis control project only followed CDTI strategy and did not deliver any vector control measures, however, a major complaint among the participants made it so important to be highlighted because it affects their work in their farms and during fishing or other activities near to the river banks, particularly the very intense biting during the harvesting time of their crops; during winter [24, 25].

Since the majority of the study participants reported high frequency of involvement in one or more festival event, this indicated high rate of social recovery in Abu-Hamed area [26–28].

## Conclusion

Apparently, the affected communities are recovering not only from the health burden of the disease but the negative social and economic impacts as well. However, still there is a long way to overcome some side effects of the disease to achieve the local developmental plans. The need for an effective vector control strategy is crucial for the development and productivity of the local communities.

## Limitations

- The study has focused mainly on the socio-economic impacts of Onchocerciasis elimination. But challenges associated with achieving the elimination and ensuring equity in all communities have not been explored.
- The insufficient data of the social and economic parameters before the beginning of the Onchocerciasis control activities in the area.

## Data Availability

All data generated or analysed during this study is presented within this manuscript.

## Abbreviations

CDTI: Community Directed Treatment with Ivermectin
mf: Microfilariae
NTDs: Neglected Tropical Diseases
SPSS: Statistical Package for Social Sciences
WHO: World Health Organization
